# Gastric Adenocarcinomas and Signet-Ring Cell Carcinoma: Unraveling Gastric Cancer Complexity through Microbiome Analysis—Deepening Heterogeneity for a Personalized Therapy

**DOI:** 10.3390/ijms21249735

**Published:** 2020-12-20

**Authors:** Gloria Ravegnini, Bruno Fosso, Viola Di Saverio, Giulia Sammarini, Federica Zanotti, Giulio Rossi, Monica Ricci, Federica D’Amico, Giorgia Valori, Antonella Ioli, Silvia Turroni, Patrizia Brigidi, Patrizia Hrelia, Sabrina Angelini

**Affiliations:** 1 Department of Pharmacy and Biotechnology, University of Bologna, 40126 Bologna, Italy; gloria.ravegnini2@unibo.it (G.R.); giulia.sammarini2@unibo.it (G.S.); federicazanotti94@gmail.com (F.Z.); federica.damico8@unibo.it (F.D.); giorgia.valori@studio.unibo.it (G.V.); silvia.turroni@unibo.it (S.T.); patrizia.hrelia@unibo.it (P.H.); 2 National Research Council, Institute of Biomembranes, Bioenergetics and Molecular Biotechnologies (IBIOM), 70126 Bari, Italy; b.fosso@ibiom.cnr.it; 3 Anatomy and Pathological Histology Unit, Infermi Hospital, 47923 Rimini, Italy; viola.ds@hotmail.it (V.D.S.); giuliorossi68@gmail.com (G.R.); monica.ricci4@auslromagna.it (M.R.); antonella.ioli@auslromagna.it (A.I.); 4 Department of Medical and Surgical Sciences, University of Bologna, 40126 Bologna, Italy; patrizia.brigidi@unibo.it

**Keywords:** gastric cancer, adenocarcinoma, ADC, signet-ring cell carcinoma, SRCC, microbiome, biomarkers, personalized therapy

## Abstract

Gastric cancer (GC) is the fifth most prevalent cancer worldwide and the third leading cause of global cancer mortality. With the advances of the omic studies, a heterogeneous GC landscape has been revealed, with significant molecular diversity. Given the multifaceted nature of GC, identification of different patient subsets with prognostic and/or predictive outcomes is a key aspect to allow tailoring of specific treatments. Recently, the involvement of the microbiota in gastric carcinogenesis has been described. To deepen this aspect, we compared microbiota composition in signet-ring cell carcinoma (SRCC) and adenocarcinoma (ADC), two distinct GC subtypes. To this purpose, 10 ADC and 10 SRCC and their paired non-tumor (PNT) counterparts were evaluated for microbiota composition through 16S rRNA analysis. Weighted and unweighted UniFrac and Bray–Curtis dissimilarity showed significant community-level separation between ADC and SRCC. Through the LEfSe (linear discriminant analysis coupled with effect size) tool, we identified potential microbial biomarkers associated with GC subtypes. In particular, SRCCs were significantly enriched in the phyla Fusobacteria, Bacteroidetes, Patescibacteria, whereas in the ADC type, Proteobacteria and Acidobacteria phyla were found. Overall, our data add new insights into GC heterogeneity and may contribute to deepening the GC classification.

## 1. Introduction

Gastric cancer (GC) is the fifth most frequently diagnosed cancer and the third cause of cancer-related death worldwide [1]. GC is a very heterogeneous disease from the histopathology, site of origin and genetic and molecular signatures standpoints [1,2,3]. The heterogeneity of GC patients is reflected by several classification systems reported over the decades; however, none of them provide reliable independent prognostic information [4,5]. In addition, patients exhibit distinct genetic and molecular profiles and comprehensive molecular analysis could help to uncover potential therapeutic targets. Despite this, there is still a question as to which classification should be followed for patient-specific management. Therefore, it is necessary to identify novel biomarkers that can unravel its heterogeneity, potentially contributing to predict survival and providing guidance for clinical treatment. Among the GC subtypes, the signet-ring cell carcinoma (SRCC) has attracted research interest as its incidence is constantly rising [6,7]. The observed SRCC increase is largely explainable by changes in the used pathological classification (for a review see ref [6,7]). In particular, SRCC is recognized as one specific histotype, defined according to the WHO’s classification as a poorly cohesive carcinoma, primarily characterized by mucin-producing cells with a crescent-shaped nucleus centrically located [7]. SRCC has distinct features compared to other GC, including adenocarcinomas (ADC). Epidemiology, clinical presentation and risk factors differ substantially; from a molecular perspective, different mutational and gene expression profiles between SRCC and ADC GC have been found, suggesting that SRCC may represent a completely distinct entity [8]. Thus, identification of the characteristics of each GC subtype is an important step in managing patients properly. Recent evidence supports the involvement of the gastrointestinal (GI) microbiota in gastric carcinogenesis [8,9,10,11]. The GI tract is a complex environment settled by trillions of microorganisms, with biogeographically delineated microbial communities that constantly communicate with each other and with the host in a symbiotic manner. The microbiota plays a key role in the human pathophysiology, acting like an additional genome, therefore affecting the health and disease status of the host. In particular, due to its multifaceted and dynamic nature, the gut microbiome has been recently considered as a metabolically active organ, supporting several processes, including but not limited to energy metabolism, pathogen elimination and cancer development. It is well recognized that the gut microbiome is an important player in etiology of GI cancers, particularly dysbiosis-associated GC. Dysbiosis, a condition characterized by GI microbiota imbalance, leads to several pathological conditions and may contribute to malignant transformation paving the way for carcinogenesis process [12,13,14,15,16,17,18,19]. Unfortunately, knowledge of the complex interactions between host and microbiota in cancer is still limited [20]. This is particularly true for the gastric microbiota, which is largely understudied compared to the intestinal counterpart [21,22]. Moreover, the majority of the studies evaluating stomach cancer have investigated the microbiome profile in mixed cohorts of GC patients without considering the different subtypes [23,24]. Consequently, the knowledge on the potential differences in microbiota communities between SRCC and ADC GC is rather absent, leaving an open gap to fulfill.

Based on the mounting evidence and the need to disentangle GC heterogeneity, we investigated the possible alterations in microbiota composition and inferred functionality in order to identify potential biomarkers to better stratify the GC in SRCC and ADC.

## 2. Results

### 2.1. Microbiota Profiles of GC and Paired Non-Tumoral Samples

We analyzed the microbiota from GC FFPE (formalin-fixed paraffin-embedded) samples, including 10 ADC and 10 SRCC; for all samples, paired non-tumor (PNT) counterparts were also investigated. For each sample, the 16S rRNA V3–V4 region analysis yielded 1,473,216 high-quality reads, corresponding to 1,562 amplicon sequence variants (ASVs). On average, we did not observe significant differences in the number of reads between tumor and normal samples (36,402.1 vs. 37,258.7, *p* = 0.899, Student’s t-test). A total of 144 ASVs were identified as belonging to mitochondrial or chloroplast ribosomal genes and were further removed from subsequent analysis.

After stratifying GC by subtypes (ADC and SRCC) and their PNT, alpha diversity was evaluated by using both Shannon and Faith’s PD (phylogenetic diversity) indexes. The analysis did not reveal significant differences in the microbial community diversity in SRCC tumor samples and matched PNT with respect to ADC tumor samples and matched PNT. In particular, the Kruskal–Wallis test showed no differences in the Shannon (*p* = 0.238) and Faith indexes (*p* = 0.679); subsequently, the Mann–Whitney test did not highlight differences between groups (Shannon and Faith indexes, respectively: ADC vs. ADC-PNT *p* = 1.00 and *p* = 0.897; SRCC vs. SRCC-PNT *p* = 0.089 and *p* = 0.270; ADC vs. SRCC *p* = 0.536 and *p* = 1; ADC-PNT vs. SRCC -PNT *p* = 0.280 and *p* = 0.529).

**Figure 1:**
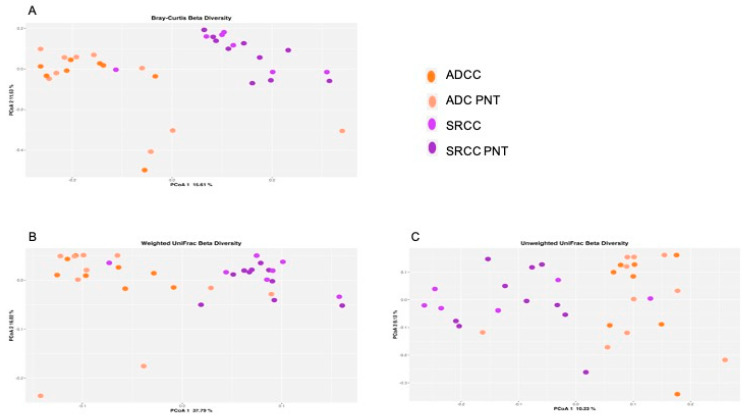
Principal coordinate analysis (PCoA) plot with Bray–Curtis dissimilarity. Results revealed significant differences between ADC (adenocarcinoma) and SRCC (signet-ring cell carcinoma) (**A**). Composition of the gut microbiota does not significantly change between ADC and SRCC samples; (**B**) unweighted and (**C**) weighted UniFrac measures of beta-diversity visualized using PCoA.

At the ASVs level, the PERMANOVA analysis revealed that the stratification of the two tumor types significantly explained the observed data variability in all the three applied metrics (i.e., Bray–Curtis dissimilarity 17% *p* = 0.001, Figure 1A; unweighted UniFrac 14% *p* = 0.001, Figure 1B; weighted UniFrac 29% *p* = 0.001, Figure 1C), whereas both SRCC and ADC clustered with their normal counterpart. In addition, we did not observe relevant variability stratification with respect to sex (Bray–Curtis dissimilarity *p* = 0.11; unweighted UniFrac *p* = 0.31; weighted UniFrac *p* = 0.6) and *Helicobacter pylori* (HP) co-infection (Bray–Curtis dissimilarity *p* = 0.45; unweighted UniFrac *p* = 0.51; weighted UniFrac *p* = 0.23).

### 2.2. The Microbiota Profiles of SRCC Tumors Differs from ADC Tumors

**Figure 2:**
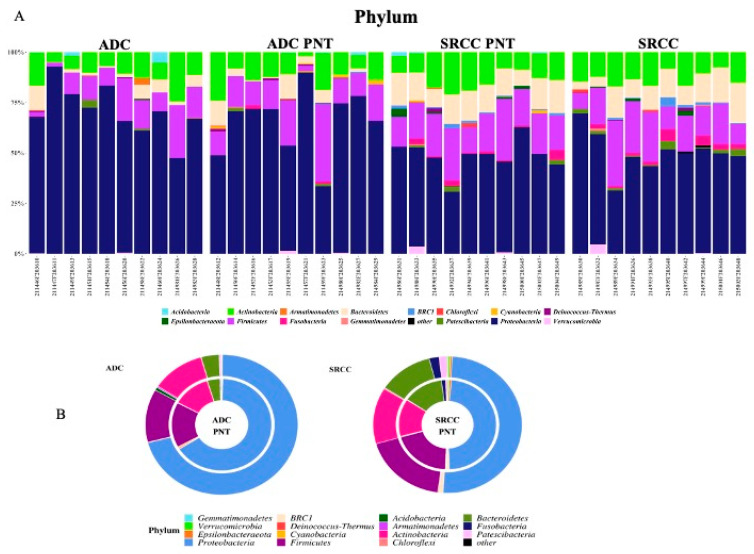
(**A**) The relative abundance at the phylum level for each sample. (**B**) Donut charts representing the relative abundance of phyla in ADC and SRCC groups. In particular, relative abundances represent the mean of the observed abundances per phylum in each group. Outer and inner donut chart represents tumor and healthy counterpart tissues, respectively.

We compared the gastric microbiota composition of ADC with SRCC tumor samples. Figure 2A,B summarize the relative abundance at the phylum level for each sample and group, respectively. Significant differences were found in the relative proportion of certain phyla; specifically, Acidobacteria *(P*_adj_ = 0.0001), Deinococcus-Thermus (*P*_adj_ = 0.0019) and BRC1 (*P*_adj_ = 0.0063) were over-represented, whereas Epsilonbacteraeota (*P*_adj_ = 0.0001) was under-represented in ADC compared to SRCC samples.

Considering the class rank, Thermoleophilia (*P*_adj_ < 0.0001), Deinococci (*P*_adj_ = 0.0016) and Bacilli (*P*_adj_ = 0.0463) were more abundant, while Campylobacteria (*P*_adj_ = 0.0001) was less abundant in ADC compared to SRCC tumor samples. At the genus level, these findings were reflected in nine genera differentially represented between the two GC subgroups, namely *Massilia*, *Aquabacterium*, *Prevotella*, *Prevotella 7*, *Dialister*, *Stenotrophomonas*, *Oribacterium*, *Halomonas* and *Shewanella*. With regard to the order, family and genus taxonomic levels, the significant results are reported in Table 1.

**Figure 3:**
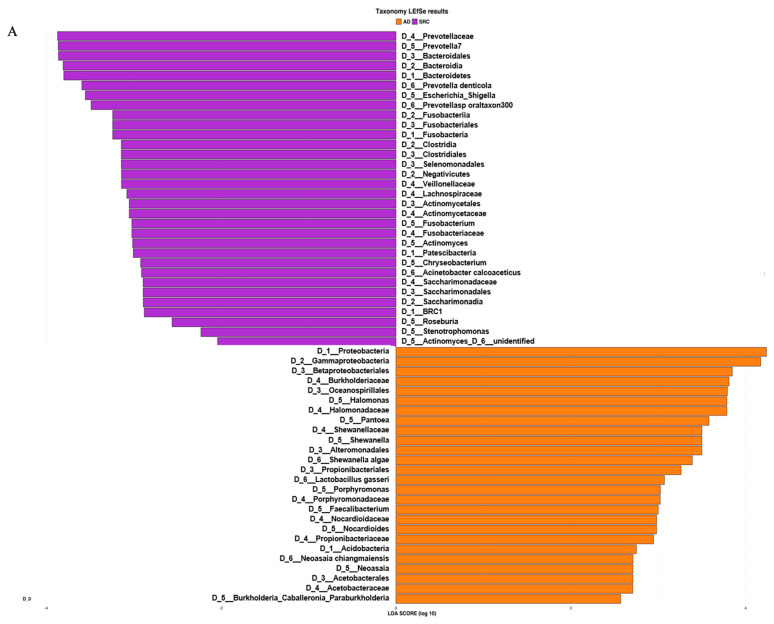

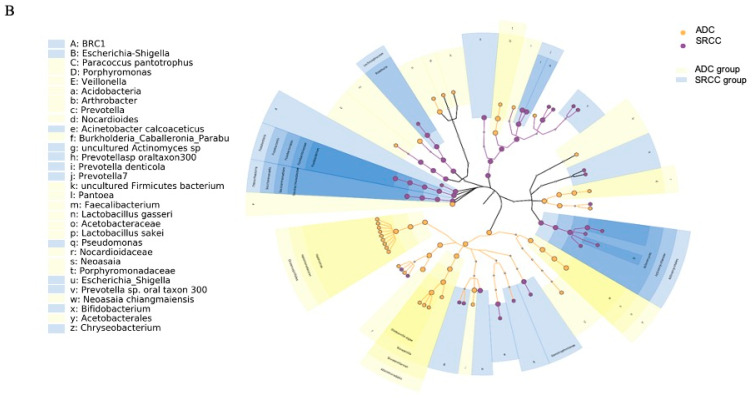
(**A**) LEfSe (linear discriminant analysis coupled with effect size) identified the most differentially abundant taxa between ADC, ADC PNT (paired non-tumor), SRCC and SRCC PNT. Only taxa meeting an LDA (linear discriminant analysis) significant threshold of >2 are shown. (**B**) Taxonomic cladogram obtained from LEfSe analysis of 16S sequences. In particular, two shapes are used to plot the nodes: circle and hexagon, corresponding to taxonomic and ASV clades, respectively. The node bodies are filled if associated to one specific condition following the LEfSe analysis. Moreover, the nodes background is imposed if all the child nodes belong to the same macro-group (i.e., ADC group and SRCC group correspond to tumor and healthy tissues collected from ADC and SRCC subjects, respectively). Unannotated clades correspond to ambiguous taxa in the reference taxonomy (i.e., SILVA).

Based on the ASV distribution, the LEfSe (linear discriminant analysis coupled with effect size) tool allowed us to identify possible microbial biomarkers associated with a specific tumor subtype (Figure 3A).

SRCC tumor samples were significantly enriched in the phyla Fusobacteria, Patescibacteria, Bacteroidetes and BRC1 and genera *Prevotella 7*, *Fusobacterium*, *Actinomyces, Stenotrophomonas* and *Roseburia*. On the other hand, the phyla Proteobacteria and Acidobacteria and the genera *Halomonas*, *Shewanella, Pantoea, Faecalibacterium* and *Neoasaia* were identified as potential biomarkers of the ADC subtype. The analysis at the ASV level allowed us to identify 27 and 20 ASVs associated with ADC and SRCC, respectively (Table S1). The ASVs associated with ADC belong to 13 genera, of which 9 were *Halomonas* and 4 *Shewanella*. In SRCC, 10 genera were observed and *Prevotella 7* was the most observed (*n* = 3). The cladogram (Figure 3B) summarizes the LEfSe association by representing the taxonomic relationship between significant ASVs associated with each group. In particular, enrichment of *Fusobacterium, Actinomyces* and *Saccharimonadaceae* were observed in SRCC cases, whereas *Halomonas and Shewanella* in ADC samples.

### 2.3. Prediction of Metabolic Functional Profile and Pathways

**Figure 4:**
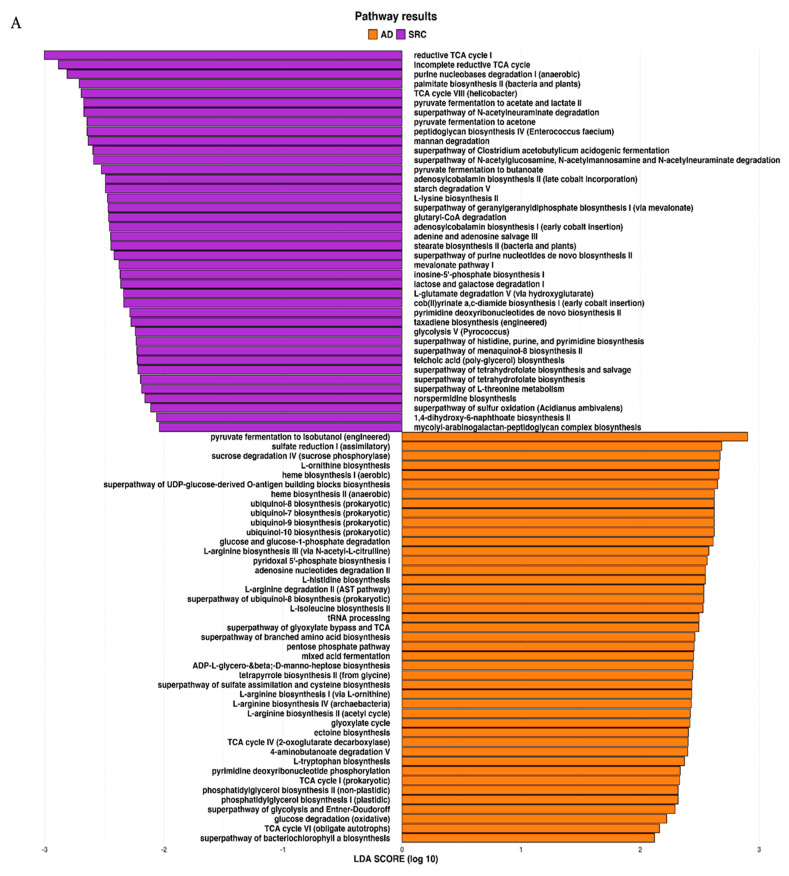

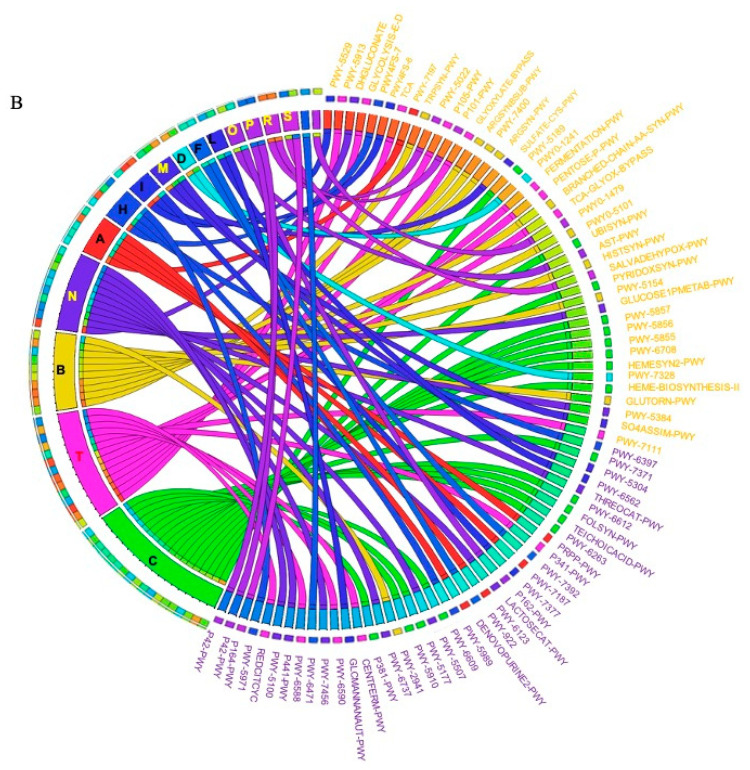
(**A**) LEfSe identified the most differentially abundant PICRUSt-predicted KEGG pathways between ADC and SRCC. SRCC-enriched pathways are indicated with a negative LDA score (purple) and pathways enriched in ADC with a positive score (orange). Only pathways meeting an LDA significant threshold of > 2 are shown. (**B**) Circos plot was generated to achieve insights into the relevant super-classes to which pathways belong.

The metagenomic functional profile, predicted with PICRUSt2, highlighted four pathways that were significantly deregulated in SRCC compared to ADC. Differentially abundant pathways, identified with the LEfSe tool, in SRCC and ADC are presented in Figure 4A. Additionally, to achieve insights into the relevant super-classes to which pathways belong, a circos-plot was generated (Figure 4B and Table S2). Both tumors were particularly enriched in biosynthesis paths, with several overlapping super-classes and some unique metabolic pathways in SRCC tumors. Degradation/utilization paths were highly represented in SRCC compared to ADC and, similarly to the biosynthesis paths, some super-classes overlap while others are unique to SRCC. Lastly, generation of precursor metabolites and energy pathways were over-identified in ADC compared to SRCC.

## 3. Discussion

GC is a heterogeneous disease affected by different genetic and environmental factors. Although *Helicobacter* (*H*.) *pylori* represents the most well-established risk factor for GC, only 1–2% of infected patients develop cancer [25,26]. This relationship between *H. pylori* and GC well describes the paradigm on how heterogeneity at the level of the microbiome and host may affect disease susceptibility and its behavior. In this context, the specific influence of additional factors on the disease and, in particular, of the microbiome, might be important in GC development. Indeed, the interaction between microbiota and host cells along the GC tract is a key player in influencing and modulating the immune system [27,28,29,30], with many reports showing changes in the microbiota composition in different inflammatory-sustained conditions [31,32]. In our study population, 50% of tumor samples were *H. pylori* positive (4 ADC and 6 SRCC) strengthening the need for further investigations. Along this perspective, we performed an omic approach aimed at identifying a set of potential markers for a better stratification in ADC or SRCC type. Indeed, knowledge on the potential differences in microbiota communities between SRCC and ADC GC is completely lacking; this is mostly due to the fact that the majority of the studies did not consider the GC subtypes and analyzed mixed cohorts of stomach cancer patients.

With the aim of characterizing the microbiota residing in the stomach tract that could actively contribute to gastric carcinogenesis, we compared the microbiome profile of 20 GC patients stratified according to the different types (i.e., ADC and SRCC). In addition, all the GC samples were compared with their matched normal counterparts; however, regarding this, we did not observe significant differences.

In our cohort of patients, Proteobacteria, Firmicutes, Actinobacteria and Bacteroidetes were the most abundant phyla in all samples, regardless of presence of the tumor (Table S3). This finding is in line with general reports which highlight Bacteroidetes and Firmicutes followed by Actinobacteria and Proteobacteria as the most abundant taxa of the intestinal microbiota of a healthy adult [33,34,35,36], although the composition and prevalence may change in cancer patients [37,38,39]. Interestingly, when we compared the two tumor types, both weighted and unweighted UniFrac and Bray–Curtis dissimilarity showed significant community-level separation between them. To the best of our knowledge, this is the first study to separately consider SRCC and ADC GC types, therefore, our data, even if preliminary, pave the way to further investigations in larger cohorts of patients. Specifically, comparing the median relative abundance profiles, we observed a pronounced difference in Proteobacteria (49.9% vs. 70.7% in SRCC vs. ADC), Firmicutes (8.4% vs. 12.2%) and Bacteroidetes (11.2% vs. 3.8%). Through the LEfSe tool we identified potential microbial biomarkers associated with a specific GC subtype. In particular, SRCC samples were significantly enriched in the phyla Fusobacteria, Bacteroidetes, Patescibacteria and BRC1. The latter is present with very low relative abundance (1% only) and no association with chronic degenerative diseases is reported in the literature. Patescibacteria members are 3.4-times higher compared to ADC, however their relative abundance is very low (1.7%) and no data are reported in the literature regarding any potential association with cancer. On the contrary, Fusobacteria and Bacteroidetes, both including anaerobic bacteria, have been shown to be enriched in cancer, including colorectal, oral and head cancers [40,41,42,43]. This is interesting, in particular with regard to Fusobacteria; indeed, considering that tumor site distribution in SRCC and ADC was similar, we can speculate that its presence does not depend on the site, but is a possible biomarker for SRCC. At the genus level, the genera *Prevotella 7* (Bacteroidetes) and *Stenotrophomonas* (Proteobacteria), confirm the results obtained by using DESeq2. The first has been recently described as a pathogen associated with nosocomial infections in immunocompromised and neonatal patients and the second has been associated with chronic gastritis [21,44].

With regard to potential biomarkers for the ADC type, Acidobacteria and Proteobacteria phyla were found. Concerning the first phylum, data are quite limited; indeed, there are no studies focused on gastric cancer. However, a recent work by Zhao et al. showed that the Acidobacteria phylum was associated with a better response to chemotherapy in lung cancer patients [45]. Therefore, further research in GC patients is strongly warranted. Regarding the second phylum, an increase in Proteobacteria, including several opportunistic pathogens and pathobionts, is commonly observed in humans with severe intestinal inflammation, including patients with inflammatory bowel disease [46,47,48]. According to Shin and coworkers, Proteobacteria could serve as a marker of microbiota instability as they are a minor component (5%) within a balanced gut-associated microbial community, while microbiota disruption (i.e., dysbiosis) generally leads to an increase in their proportion [49]. Among the genera associated with ADC, the halophilic *Halomonas* and *Shewanella* (both Proteobacteria) were the most represented. Both have been already described in GC microbiomes [22,50], probably due to their ability to colonize the stomach once gastric secretion is reduced. However, the result is quite attractive, as a recent study by Ren et al., performed on Asian patients, reported a high abundance of these bacteria in patients with gastric polyps compared to healthy controls [50]. In this work, the authors hypothesized that the abundance of halophilic bacteria could be related to the high consumption of preserved or salty food [50]. Thus, by relating the hypothesis of Ren and co-workers with our data, we could hypothesize that a diet promoting halophilic bacteria may be a risk factor for GC and in particular for the ADC type. With regard to metabolic prediction, a high number of pathways involving L-arginine biosynthesis were observed in ADC, but not in SRCC. Arginine is pivotal for the growth of human cancer and in regulating tumor metabolism, including the synthesis of nitric oxide (NO), polyamines, nucleotides, proline and glutamate. Although arginine is not an essential amino acid, it enhances tumor growth and its restriction inhibits the growth of metastatic tumors [51,52]. Confirming the importance of this amino acid, some tumors, including hepatocellular carcinoma and melanoma, are auxotrophic to arginine. Interestingly, a work by Kim et al. performed on a cell model of ADC demonstrated that arginine depletion induces G1-phase cell cycle arrest and apoptosis [53]. This finding, together with our data, suggests that ADC cells are auxotrophic to arginine; however, tumors can also rely on external sources, including bacteria, to obtain nutrients, including arginine. A growing emphasis has been given to the NO pathway, as it profoundly affects physiological function, and, in turn, an imbalance in its levels may promote cancer [54]. In view of these considerations, targeting the NO signaling might be of major importance as a therapeutic target in ADC compared to SRCC. With regard to SRCC, we observed a higher number of inferred metabolic pathways involved in degradation (13 paths versus 8, in SRCC and ADC, respectively), with three of those belonging to carboxylate degradation and two related to C1 compound utilization and assimilation, which were not found in ADC. Three unique classes of pathways related to biosynthesis were also observed in SRCC. Among them, we found the class belonging to pyrimidine biosynthesis, which could represent a metabolic target for this GC subtypes. Interestingly, pyrimidine metabolism pathways have been correlated with progression of breast and lung cancer in previous studies [55]. Therefore, its presence in SRCC alone could be related to the poorer prognosis usually associated with SRCC compared to ADC [56].

Over the past decades, advances in sequencing technologies and high-throughput analysis have delivered new insights into the genetic heterogeneity that underlies the distinct GC molecular subtypes [57,58,59]. Unfortunately, with regard to microbiota, no other studies have previously investigated SRCC and ADC GC types as distinct populations, thus we cannot compare our data with other evidence. In general, our results highlighted specific differences in microbiome prevalence between SRCC and ADC; this is in agreement with molecular analysis at the genetic and epigenetic level which highlighted specific profiles based on the GC subtype [60,61].

We are aware that from a technical point of view FFPE could not represent the ideal specimen for microbiome studies. Since formalin is recognized to affect DNA quality, this may have limited our capability to detect bacteria. However, considering that all samples were handled equally, we do not expect the formalin fixation to impact the observed differences in bacterial load and prevalence between groups, as previously reported [62]. Nevertheless, given the limited availability of fresh/frozen samples, unravelling the potential of FFPE samples for microbiome analysis could have a massive effect on the field. Our work shows that FFPE could be used a starting material to analyze microbiome communities in oncological patients.

## 4. Material and Methods

### 4.1. Samples Collection

A total of 20 GC patients, all of Caucasian ethnicity, were included in this study. All surgical specimens were reviewed at multiheaded microscope by two pathologists (GR, MR) and a consensus diagnosis was established according to the most recent World Health Organization classification of gastric tumors [63]. For every patient, both tumor and PNT FFPE samples were available from the same surgical procedure, collected in different FFPE blocks; tumor FFPE were confirmed SRCC and ADC phenotypes. Tissue was defined normal (non-tumor tissue) by three different pathologists who evaluated independently the material.

All patients were resected at the Infermi Hospital, between 2009 and 2015. The study was approved by the Wide Catchment Area of Romagna (AVR) Scientific Medical committee (protocol number 0006002/2017). Table 2 summarizes the main clinical characteristics of the GC patients.

### 4.2. 16S rRNA Sequencing and Bioinformatics

Total DNA of 40 FFPE samples from 20 GC patients was isolated using RecoverAll Total Nucleic Acid Isolation Kit (ThermoFisher Scientific, Waltham, MA, USA), according to the manufacturer’s protocol. In particular, 10 samples were ADC, 10 were SRCC and 20 were matched non-tumor samples. Briefly, for microbiome analysis, the V3–V4 hypervariable regions of the 16S rRNA gene were amplified by using universal primer pairs [64] with Illumina overhang adapter sequences. PCR products of ~460 bp were purified using a magnetic bead-based, indexed by limited-cycle PCR using Nextera technology and were additionally purified using Agencourt AMPure XP magnetic beads. Indexed libraries were pooled at an equimolar concentration, denatured and diluted before loading onto the MiSeq flow cell. Sequencing was carried out on an Illumina MiSeq platform using a 2 × 300 bp paired-end approach. Sequencing reads were deposited in the National Center for Biotechnology Information Sequence Read Archive (NCBI SRA; BioProject ID PRJNA641258).

Raw sequence data were denoised and ASVs were inferred by applying DADA2 (v1.10.1) [65,66]. Following the ASVs inference, human contaminants were identified by using bowtie2 (v2.3.4.1) [67] and the hg19 assembly of the human genome. ASVs were taxonomically classified by using the QIIME2 [68] plugin classify-sklearn [69] and the release 132 of the SILVA database [70]. A rooted phylogenetic tree based on ASV sequences was obtained by applying the QIIME2 plugin align-to-tree-mafft-fasttree [71,72]. The metagenome functional profile and in particular functional pathways were predicted by using PICRUSt2 [73]. In particular, PICRUSt2 places the inferred ASV sequences on a reference phylogenetic tree collecting about 20,000 full 16S rRNA sequences from prokaryotic genomes. The ASVs placement allows the prediction of genes content and the normalization of it according to ASVs abundances. Finally, functional pathways were inferred by using MinPath [74] upon the KEGG [75] database annotations. Alpha and beta diversity analysis were performed by using the R packages Phyloseq (v1.26.1) [76] and Vegan (v2.5–6) [77]. For this purpose, ASVs counts and pathways were normalized by using rarefaction. The Shannon and the Faith’s phylogenetic diversity (PD) indices were inferred to measure the intra-samples diversity (alpha diversity). The non-parametric Kruskal–Wallis and Mann–Whitney statistical tests were performed to compare the alpha diversity distributions between conditions. Differences in community structure between samples (beta diversity) were evaluated using the Bray–Curtis dissimilarity index and weighted and unweighted UniFrac phylogenetic metrics [78]. Permutational Multivariate Analysis of Variance Using Distance Matrices (PERMANOVA, 999 permutations) was computed using the Adonis function of the Vegan package by considering sample type, sex and their interaction. In order to identify statistically significant taxa and functions, normalized counts were compared using DESeq2 [79] by considering sample type, sex, age and sampling site. The obtained *p*-values were normalized using the Benjamini–Hochberg formula; a corrected *P_adj_* < 0.05 was considered statistically significant. Finally, ASVs and pathways associated with a specific status (i.e., ADC vs. SRCC) were identified by using linear discriminant analysis (LDA) coupled with effect size (LEfSe) [80]. LDA scores (log 10) ≥ 2 were retained.

## 5. Conclusions

In conclusion, our data add new insights into GC heterogeneity, serving as a foundation for future research directed at a better diagnosis and precision treatment. Indeed, our data showed differences in the prevalence of microbiota communities in GC. Furthermore, at the same time, we found significant differences between the two tumor subtypes, ADC and SRCC, which have not been previously described and therefore are potentially useful to provide additional information on prognosis. We are fully aware that the study is preliminary, and our conclusions are limited by the lack of proper healthy controls, a replication dataset and the small sample size; therefore, findings will require confirmation in further studies. Nevertheless, our findings, if confirmed by further studies, may contribute to deepening the GC heterogeneity and its classification scheme, which may facilitate personalized medicine in ADC and SRCC.

## Figures and Tables

**Table 1:** Difference in abundance in microbial communities at the phylum, class, order, family and genus taxonomic ranks.

**Phylum**	**FC (ADC vs. SRCC)**	**padj**
D_1__Epsilonbacteraeota	−13.75	0.0001
D_1__Acidobacteria	16.97	0.0001
D_1__Deinococcus-Thermus	16.06	0.0019
D_1__BRC1	8.97	0.0063
**Class**	**FC**	**padj**
D_1__Actinobacteria;D_2__Thermoleophilia	29.26	0.0000
D_1__Deinococcus-Thermus;D_2__Deinococci	16.11	0.0016
D_1__Firmicutes;D_2__Bacilli	−1.02	0.0463
D_1__Epsilonbacteraeota;D_2__Campylobacteria	−13.82	0.0001
**Order**	**FC**	**padj**
D_1__Bacteroidetes;D_2__Bacteroidia;D_3__Cytophagales	18.32	0.0009
D_1__Deinococcus-Thermus;D_2__Deinococci;D_3__Deinococcales	16.25	0.0012
D_1__Proteobacteria;D_2__Gammaproteobacteria;D_3__Oceanospirillales	6.08	0.0237
D_1__Proteobacteria;D_2__Gammaproteobacteria;D_3__Alteromonadales	6.04	0.0095
D_1__Proteobacteria;D_2__Alphaproteobacteria;D_3__Sphingomonadales	4.18	0.0095
D_1__Actinobacteria;D_2__Actinobacteria;D_3__Propionibacteriales	1.69	0.0095
D_1__Actinobacteria;D_2__Actinobacteria;D_3__Bifidobacteriales	−5.15	0.0241
D_1__Bacteroidetes;D_2__Bacteroidia;D_3__Bacteroidales	−5.46	0.0009
D_1__Epsilonbacteraeota;D_2__Campylobacteria;D_3__Campylobacterales	−12.84	0.0009
**Family**	**FC**	**padj**
D_1__Proteobacteria;D_2__Gammaproteobacteria;D_3__Oceanospirillales;D_4__Halomonadaceae	5.92	0.0468
D_1__Proteobacteria;D_2__Alphaproteobacteria;D_3__Sphingomonadales;D_4__Sphingomonadaceae	3.78	0.0415
D_1__Actinobacteria;D_2__Actinobacteria;D_3__Bifidobacteriales;D_4__Bifidobacteriaceae	−5.48	0.0415
D_1__Firmicutes;D_2__Clostridia;D_3__Clostridiales;D_4__Lachnospiraceae	−5.72	0.0342
D_1__Bacteroidetes;D_2__Bacteroidia;D_3__Bacteroidales;D_4__Prevotellaceae	−7.18	0.0003
D_1__Firmicutes;D_2__Clostridia;D_3__Clostridiales;D_4__Peptostreptococcaceae	−10.45	0.0415
**Genus**	**FC**	**padj**
D_1__Proteobacteria;D_2__Gammaproteobacteria;D_3__Betaproteobacteriales;D_4__Burkholderiaceae;D_5__Aquabacterium	22.47	0.0000
D_1__Proteobacteria; D_2__Gammaproteobacteria; D_3__Betaproteobacteriales; D_4__Burkholderiaceae; D_5__Massilia	17.23	0.0000
D_1__Proteobacteria;D_2__Gammaproteobacteria;D_3__Alteromonadales;D_4__Shewanellaceae;D_5__Shewanella	5.93	0.0327
D_1__Proteobacteria;D_2__Gammaproteobacteria;D_3__Oceanospirillales;D_4__Halomonadaceae;D_5__Halomonas	5.72	0.0284
“D_0__Bacteria;D_1__Bacteroidetes;D_2__Bacteroidia;D_3__Bacteroidales;D_4__Prevotellaceae;D_5__Prevotella 7	−6.71	0.0020
D_1__Proteobacteria;D_2__Gammaproteobacteria;D_3__Xanthomonadales;D_4__Xanthomonadaceae;D_5__Stenotrophomonas	−11.66	0.0159
D_1__Firmicutes;D_2__Clostridia;D_3__Clostridiales;D_4__Lachnospiraceae;D_5__Oribacterium	−11.75	0.0200
D_1__Bacteroidetes;D_2__Bacteroidia;D_3__Bacteroidales;D_4__Prevotellaceae;D_5__Prevotella	−12.50	0.0003
D_1__Firmicutes;D_2__Negativicutes;D_3__Selenomonadales;D_4__Veillonellaceae;D_5__Dialister	−16.08	0.0019

FC: Fold Change.

**Table 2:** Summary of the patients’ characteristics.

# Patient	Subgroup	Grade	Site	HP	Sex	Age at Diagnosis	Other Characteristics
6	ADC	G3	angulus	−	M	65	/
11	ADC	G3	antrum-pylorus	+	M	67	chronic gastritis inflammatory infiltrate
12	ADC	G4	antrum	−	F	61	chronic atrophic gastritis with intestinal metaplasia
25	ADC	G3	antrum -Body	−	F	68	/
26	ADC	G3	Body	+	F	81	chronic atrophic gastritis with intestinal metaplasia
33	ADC	G3	angulus	−	F	82	chronic atrophic gastritis with intestinal metaplasia
37	ADC	G3	antrum	+	F	85	chronic gastritis
42	ADC	G3	cardias	−	F	80	chronic atrophic gastritis with intestinal metaplasia
46	ADC	G3	antrum	+	F	71	chronic atrophic gastritis with intestinal metaplasia
52	ADC	G3	angulus	−	F	82	chronic active gastritis
18	SRCC	G3	pylorus	−	M	81	chronic gastritis
20	SRCC	G4	antrum-Body	+	M	72	chronic atrophic gastritis with intestinal metaplasia
36	SRCC	G3	antrum-Body	+	F	82	chronic atrophic gastritis with intestinal metaplasia
39	SRCC	G3	antrum	+	F	89	chronic atrophic gastritis with intestinal metaplasia
41	SRCC	G4	angulus	−	M	69	chronic atrophic gastritis with intestinal metaplasia
43	SRCC	G3	antrum-pylorus	−	F	72	chronic atrophic gastritis with intestinal metaplasia
44	SRCC	G3	antrum-piloro	+	M	61	chronic gastritis
45	SRCC	G4	antrum	+	M	85	chronic atrophic gastritis with intestinal metaplasia
47	SRCC	G3	antrum-pylorus	+	M	58	chronic gastritis
48	SRCC	G3	angulus	−	M	77	/

−: negative; +: positive; /: missing information.

## Supplementary Materials

The following are available online at https://www.mdpi.com/1422-0067/21/24/9735/s1, Table S1: LefSe analysis at the ASV level, Table S2: Details of pathways reported in the circos-plot, Table S3: Relative abundance in microbial communities in SRCC, SRCC PNT, ADC and ADC PNT samples.

## Abbreviations

GC	Gastric cancer
SRCC	Signet-ring cell carcinoma
ADC	Adenocarcinoma
PNT	Paired non-tumor
ASV	Amplicon sequence variants
GI	Gastrointestinal
FFPE	Formalin-Fixed Paraffin-Embedded
PCoA	Principal coordinate analysis
PD	Phylogenetic Diversity
LEfSe	Linear discriminant analysis Effect Size
LDA	Linear Discriminant Analysis

## References

[1] Razzak M. (2014). New molecular classification of gastric adenocarcinoma proposed by The Cancer Genome Atlas. Nat. Rev. Gastroenterol. Hepatol..

[2] Shah M.A., Khanin R., Tang L., Janjigian Y.Y., Klimstra D.S., Gerdes H., Kelsen D.P. (2011). Molecular Classification of Gastric Cancer: A New Paradigm. Clin. Cancer Res..

[3] Bass A.J., Thorsson V., Shmulevich I., Reynolds S.M., Miller M., Bernard B., Hinoue T., Laird P.W., Curtis C., Shen H. (2014). Comprehensive molecular characterization of gastric adenocarcinoma. Nature.

[4] Gullo I., Carneiro F., Oliveira C., Almeida G.M. (2018). Heterogeneity in Gastric Cancer: From Pure Morphology to Molecular Classifications. Pathobiology.

[5] Lim B., Kim J.-H., Kim M., Kim S.-Y. (2016). Genomic and epigenomic heterogeneity in molecular subtypes of gastric cancer. World J. Gastroenterol..

[6] Machlowska J., Pucułek M., Sitarz M., Terlecki P., Maciejewski R., Sitarz R. (2019). State of the art for gastric signet ring cell carcinoma: From classification, prognosis, and genomic characteristics to specified treatments. Cancer Manag. Res..

[7] Pernot S., Voron T., Perkins G., Lagorce-Pages C., Berger A., Taieb J. (2015). Signet-ring cell carcinoma of the stomach: Impact on prognosis and specific therapeutic challenge. World J. Gastroenterol..

[8] Taghavi S., Jayarajan S.N., Davey A., Willis A.I. (2012). Prognostic significance of signet ring gastric cancer. J. Clin. Oncol..

[9] Abreu M.T., Peek R.M. (2014). Gastrointestinal malignancy and the microbiome. Gastroenterology.

[10] Wang L., Zhou J., Xin Y., Geng C., Tian Z., Yu X., Dong Q. (2016). Bacterial overgrowth and diversification of microbiota in gastric cancer. Eur. J. Gastroenterol. Hepatol..

[11] Hu Y.-L., Pang W., Huang Y., Zhang Y., Zhang C.-J. (2018). The Gastric Microbiome Is Perturbed in Advanced Gastric Adenocarcinoma Identified Through Shotgun Metagenomics. Front. Cell. Infect. Microbiol..

[12] Gunathilake M.N., Lee J., Choi I.J., Kim Y., Ahn Y., Park C., Kim J. (2019). Association between the relative abundance of gastric microbiota and the risk of gastric cancer: A case-control study. Sci. Rep..

[13] Cho I., Blaser M.J. (2012). The human microbiome: At the interface of health and disease. Nat. Rev. Genet..

[14] Turnbaugh P.J., Ley R.E., Hamady M., Fraser-Liggett C.M., Knight R., Gordon J.I. (2007). The Human Microbiome Project. Nature.

[15] García-Castillo V., Sanhueza E., McNerney E., Onate S.A., García A. (2016). Microbiota dysbiosis: A new piece in the understanding of the carcinogenesis puzzle. J. Med. Microbiol..

[16] Gilbert J.A., Blaser M.J., Caporaso J.G., Jansson J.K., Lynch S.V., Knight R. (2018). Current understanding of the human microbiome. Nat. Med..

[17] Proctor L.M., Creasy H.H., Fettweis J.M., Lloyd-Price J., Mahurkar A., Zhou W., Buck G.A., Snyder M.P., Strauss J.F., Weinstock G.M. (2019). The Integrative Human Microbiome Project. Nature.

[18] Garrett W.S. (2015). Cancer and the microbiota. Science.

[19] Helmink B.A., Khan M.A.W., Hermann A., Gopalakrishnan V., Wargo J.A. (2019). The microbiome, cancer, and cancer therapy. Nat. Med..

[20] Schroeder B.O., Bäckhed F. (2016). Signals from the gut microbiota to distant organs in physiology and disease. Nat. Med..

[21] Ferreira R.M., Pereira-Marques J., Pinto-Ribeiro I., Costa J.L., Carneiro F., MacHado J.C., Figueiredo C. (2018). Gastric microbial community profiling reveals a dysbiotic cancer-associated microbiota. Gut.

[22] Liu X., Shao L., Liu X., Ji F., Mei Y., Cheng Y., Liu F., Yan C., Li L., Ling Z. (2019). Alterations of gastric mucosal microbiota across different stomach microhabitats in a cohort of 276 patients with gastric cancer. EBioMedicine.

[23] Chen X.H., Wang A., Chu A.N., Gong Y.H., Yuan Y. (2019). Mucosa-associated microbiota in gastric cancer tissues compared with non-cancer tissues. Front. Microbiol..

[24] Wang L., Liu J., Yu X., Zhai X., Dong Q. (2018). Microbial community reshaped in gastric cancer. Eur. Rev. Med. Pharmacol. Sci..

[25] Herrera V., Parsonnet J. (2009). Helicobacter pylori and gastric adenocarcinoma. Clin. Microbiol. Infect..

[26] Hsu P.I., Lai K.H., Hsu P.N., Lo G.H., Yu H.C., Chen W.C., Tsay F.W., Lin H.C., Tseng H.H., Ger L.P. (2007). Helicobacter pylori infection and the risk of gastric malignancy. Am. J. Gastroenterol..

[27] Grigg J.B., Sonnenberg G.F. (2017). Host-Microbiota Interactions Shape Local and Systemic Inflammatory Diseases. J. Immunol..

[28] Slingerland A.E., Schwabkey Z., Wiesnoski D.H., Jenq R.R. (2017). Clinical evidence for the microbiome in inflammatory diseases. Front. Immunol..

[29] Honda K., Littman D.R. (2016). The microbiota in adaptive immune homeostasis and disease. Nature.

[30] Thaiss C.A., Zmora N., Levy M., Elinav E. (2016). The microbiome and innate immunity. Nature.

[31] Lloyd-Price J., Arze C., Ananthakrishnan A.N., Schirmer M., Avila-Pacheco J., Poon T.W., Andrews E., Ajami N.J., Bonham K.S., Brislawn C.J. (2019). Multi-omics of the gut microbial ecosystem in inflammatory bowel diseases. Nature.

[32] Clemente J.C., Manasson J., Scher J.U. (2018). The role of the gut microbiome in systemic inflammatory disease. BMJ.

[33] Yu G., Torres J., Hu N., Medrano-Guzman R., Herrera-Goepfert R., Humphrys M.S., Wang L., Wang C., Ding T., Ravel J. (2017). Molecular characterization of the human stomach microbiota in Gastric Cancer Patients. Front. Cell. Infect. Microbiol..

[34] Greenhalgh K., Meyer K.M., Aagaard K.M., Wilmes P. (2016). The human gut microbiome in health: Establishment and resilience of microbiota over a lifetime. Environ. Microbiol..

[35] Huttenhower C., Gevers D., Knight R., Abubucker S., Badger J.H., Chinwalla A.T., Creasy H.H., Earl A.M., Fitzgerald M.G., Fulton R.S. (2012). Structure, function and diversity of the healthy human microbiome. Nature.

[36] Vivarelli S., Salemi R., Candido S., Falzone L., Santagati M., Stefani S., Torino F., Banna G.L., Tonini G., Libra M. (2019). Gut Microbiota and Cancer: From Pathogenesis to Therapy. Cancers.

[37] Sommer F., Bäckhed F. (2013). The gut microbiota-masters of host development and physiology. Nat. Rev. Microbiol..

[38] Arumugam M., Raes J., Pelletier E., Le Paslier D., Yamada T., Mende D.R., Fernandes G.R., Tap J., Bruls T., Batto J.M. (2011). Enterotypes of the human gut microbiome. Nature.

[39] Lozupone C.A., Stombaugh J.I., Gordon J.I., Jansson J.K., Knight R. (2012). Diversity, stability and resilience of the human gut microbiota. Nature.

[40] Yeoh Y.K., Chen Z., Wong M.C.S., Hui M., Yu J., Ng S.C., Sung J.J.Y., Chan F.K.L., Chan P.K.S. (2020). Southern Chinese populations harbour non-nucleatum Fusobacteria possessing homologues of the colorectal cancer-associated FadA virulence factor. Gut.

[41] Bronzato J.D., Bomfim R.A., Edwards D.H., Crouch D., Hector M.P., Gomes B.P.F.A. (2020). Detection of Fusobacterium in oral and head and neck cancer samples: A systematic review and meta-analysis. Arch. Oral Biol..

[42] Yang C.Y., Yeh Y.M., Yu H.Y., Chin C.Y., Hsu C.W., Liu H., Huang P.J., Hu S.N., Liao C.T., Chang K.P. (2018). Oral microbiota community dynamics associated with oral squamous cell carcinoma staging. Front. Microbiol..

[43] Falony G., Joossens M., Vieira-Silva S., Wang J., Darzi Y., Faust K., Kurilshikov A., Bonder M.J., Valles-Colomer M., Vandeputte D. (2016). Population-level analysis of gut microbiome variation. Science.

[44] Mutlu M., Yilmaz G., Aslan Y., Bayramoĝlu G. (2011). Risk factors and clinical characteristics of Stenotrophomonas maltophilia infections in neonates. J. Microbiol. Immunol. Infect..

[45] Zhao Z., Fei K., Bai H., Wang Z., Duan J., Wang J. (2020). Metagenome association study of the gut microbiome revealed biomarkers linked to chemotherapy outcomes in locally advanced and advanced lung cancer. Thorac. Cancer.

[46] Litvak Y., Byndloss M.X., Tsolis R.M., Bäumler A.J. (2017). Dysbiotic Proteobacteria expansion: A microbial signature of epithelial dysfunction. Curr. Opin. Microbiol..

[47] Liu C.J., Zhang Y.L., Shang Y.U.N., Wu B., Yang E.N., Luo Y.Y., Li X.R. (2019). Intestinal bacteria detected in cancer and adjacent tissue from patients with colorectal cancer. Oncol. Lett..

[48] Ma J., Sun L., Liu Y., Ren H., Shen Y., Bi F., Zhang T., Wang X. (2020). Alter between gut bacteria and blood metabolites and the anti-tumor effects of Faecalibacterium prausnitzii in breast cancer. BMC Microbiol..

[49] Shin N.R., Whon T.W., Bae J.W. (2015). Proteobacteria: Microbial signature of dysbiosis in gut microbiota. Trends Biotechnol..

[50] Ren R., Wang Z., Sun H., Gao X., Sun G., Peng L., Yan B., Yang Y. (2018). The gastric mucosal-associated microbiome in patients with gastric polyposis. Sci. Rep..

[51] Gonzalez G.G., Byus C.V. (1991). Effect of Dietary Arginine Restriction upon Ornithine and Polyamine Metabolism during Two-Stage Epidermal Carcinogenesis in the Mouse. Cancer Res..

[52] Wu G., Morris S.M. (1998). Arginine metabolism: Nitric oxide and beyond. Biochem. J..

[53] Kim J.E., Kim S.Y., Lee K.W., Lee H.J. (2009). Arginine deiminase originating from Lactococcus lactis ssp. lactis American Type Culture Collection (ATCC) 7962 induces G1-phase cell-cycle arrest and apoptosis in SNU-1 stomach adenocarcinoma cells. Br. J. Nutr..

[54] Lundberg J.O., Weitzberg E. (2013). Biology of nitrogen oxides in the gastrointestinal tract. Gut.

[55] Cheng C., Wang Z., Wang J., Ding C., Sun C., Liu P., Xu X., Liu Y., Chen B., Gu B. (2020). Characterization of the lung microbiome and exploration of potential bacterial biomarkers for lung cancer. Transl. Lung Cancer Res..

[56] Tang C.T., Chen Y., Zeng C. (2020). Prognostic analysis of gastric signet ring cell carcinoma and mucinous carcinoma: A propensity score-matched study and competing risk analysis. Aging.

[57] Zang Z.J., Cutcutache I., Poon S.L., Zhang S.L., McPherson J.R., Tao J., Rajasegaran V., Heng H.L., Deng N., Gan A. (2012). Exome sequencing of gastric adenocarcinoma identifies recurrent somatic mutations in cell adhesion and chromatin remodeling genes. Nat. Genet..

[58] Wong S.S., Kim K.M., Ting J.C., Yu K., Fu J., Liu S., Cristescu R., Nebozhyn M., Gong L., Yue Y.G. (2014). Genomic landscape and genetic heterogeneity in gastric adenocarcinoma revealed by whole-genome sequencing. Nat. Commun..

[59] Kakiuchi M., Nishizawa T., Ueda H., Gotoh K., Tanaka A., Hayashi A., Yamamoto S., Tatsuno K., Katoh H., Watanabe Y. (2014). Recurrent gain-of-function mutations of RHOA in diffuse-type gastric carcinoma. Nat. Genet..

[60] Yang M., Kim H.S., Cho M.Y. (2014). Different methylation profiles between intestinal and diffuse sporadic gastric carcinogenesis. Clin. Res. Hepatol. Gastroenterol..

[61] Jin S., Xu B., Yu L., Fu Y., Wu H., Fan X., Wei J., Liu B. (2017). The PD-1, PD-L1 expression and CD3+ T cell infiltration in relation to outcome in advanced gastric signet-ring cell carcinoma, representing a potential biomarker for immunotherapy. Oncotarget.

[62] Bundgaard-Nielsen C., Baandrup U.T., Nielsen L.P., Sørensen S. (2019). The presence of bacteria varies between colorectal adenocarcinomas, precursor lesions and non-malignant tissue. BMC Cancer.

[63] Nagtegaal I.D., Odze R.D., Klimstra D., Paradis V., Rugge M., Schirmacher P., Washington K.M., Carneiro F., Cree I.A. (2020). The 2019 WHO classification of tumours of the digestive system. Histopathology.

[64] Klindworth A., Pruesse E., Schweer T., Peplies J., Quast C., Horn M., Glöckner F.O. (2013). Evaluation of General 16S Ribosomal RNA Gene PCR Primers for Classical and Next-Generation Sequencing-Based Diversity Studies. Nucleic Acids Res..

[65] Callahan B.J., McMurdie P.J., Holmes S.P. (2017). Exact sequence variants should replace operational taxonomic units in marker-gene data analysis. ISME J..

[66] Callahan B.J., McMurdie P.J., Rosen M.J., Han A.W., Johnson A.J.A., Holmes S.P. (2016). DADA2: High-resolution sample inference from Illumina amplicon data. Nat. Methods.

[67] Langmead B., Salzberg S.L. (2012). Fast gapped-read alignment with Bowtie 2. Nat. Methods.

[68] Bolyen E., Rideout J.R., Dillon M.R., Bokulich N.A., Abnet C.C., Al-Ghalith G.A., Alexander H., Alm E.J., Arumugam M., Asnicar F. (2019). Reproducible, interactive, scalable and extensible microbiome data science using QIIME 2. Nat. Biotechnol..

[69] Abraham A., Pedregosa F., Eickenberg M., Gervais P., Mueller A., Kossaifi J., Gramfort A., Thirion B., Varoquaux G. (2014). Machine learning for neuroimaging with scikit-learn. Front. Neuroinform..

[70] Pruesse E., Quast C., Knittel K., Fuchs B.M., Ludwig W., Peplies J., Glöckner F.O. (2007). SILVA: A comprehensive online resource for quality checked and aligned ribosomal RNA sequence data compatible with ARB. Nucleic Acids Res..

[71] Katoh K., Standley D.M. (2013). MAFFT multiple sequence alignment software version 7: Improvements in performance and usability. Mol. Biol. Evol..

[72] Price M.N., Dehal P.S., Arkin A.P. (2010). FastTree 2-approximately maximum-likelihood trees for large alignments. PLoS ONE.

[73] Douglas G.M., Maffei V.J., Zaneveld J.R., Yurgel S.N., Brown J.R., Taylor C.M., Huttenhower C., Langille M.G.I. (2020). PICRUSt2 for prediction of metagenome functions. Nat. Biotechnol..

[74] Ye Y., Doak T.G. (2009). A parsimony approach to biological pathway reconstruction/inference for genomes and metagenomes. PLoS Comput. Biol..

[75] Kanehisa M., Goto S. (2000). KEGG: Kyoto encyclopedia of genes and genomes. Nucleic Acids Res..

[76] McMurdie P.J., Holmes S. (2013). Phyloseq: An R package for reproducible interactive analysis and graphics of microbiome census data. PLoS ONE.

[77] Oksanen J., Blanchet F.G., Kindt R., Legendre P., Minchin P.R. (2012). Vegan: Community Ecology Package; R Package Version 2.0–2.

[78] Chang Q., Luan Y., Sun F. (2011). Variance adjusted weighted UniFrac: A powerful beta diversity measure for comparing communities based on phylogeny. BMC Bioinform..

[79] Love M.I., Huber W., Anders S. (2014). Moderated estimation of fold change and dispersion for RNA-seq data with DESeq2. Genome Biol..

[80] Segata N., Izard J., Waldron L., Gevers D., Miropolsky L., Garrett W.S., Huttenhower C. (2011). Metagenomic biomarker discovery and explanation. Genome Biol..

