# Supplementary material for: Gastric Adenocarcinomas and Signet-Ring Cell Carcinoma: Unraveling Gastric Cancer Complexity through Microbiome Analysis—Deepening Heterogeneity for a Personalized Therapy
Source: Int J Mol Sci. 2020 Dec 20;21(24):9735. doi: 10.3390/ijms21249735 (PMC7766162; doi:10.3390/ijms21249735)
Supplement: Supplementary file 1 [file ijms-21-09735-s001.pdf]

Table S1. LefSe Analysis at the ASV level

ASV	Class	LDA score	Taxonomy
24a16ae880ca8034b6fe14f472495a22	ADC	3.62	D_1__Proteobacteria;D_2__Gammaproteobacteria;D_3__Oceanospirillales;D_4__Halomonadaceae;D_5__Halomonas
b26cfc222f563e575670d67eedcde614	ADC	2.71	D_1__Proteobacteria;D_2__Gammaproteobacteria;D_3__Oceanospirillales;D_4__Halomonadaceae;D_5__Halomonas
723ec7f372431516614f6a732b6f6bfe	ADC	3.82	D_1__Proteobacteria;D_2__Gammaproteobacteria;D_3__Oceanospirillales;D_4__Halomonadaceae;D_5__Halomonas
acaeb04ad028da418601c7243bcf363a	ADC	2.92	D_1__Proteobacteria;D_2__Gammaproteobacteria;D_3__Alteromonadales;D_4__Shewanellaceae;D_5__Shewanella;D_6__Shewanella algae
b55aafe7de7a719188448596bfd5353b	ADC	3.30	D_1__Firmicutes;D_2__Bacilli;D_3__Lactobacillales;D_4__Lactobacillaceae;D_5__Lactobacillus;D_6__uncultured Firmicutes bacterium
c4c0426b4378ca0004830ac51e9de9a5	ADC	4.63	D_1__Proteobacteria;D_2__Gammaproteobacteria;D_3__Enterobacteriales;D_4__Enterobacteriaceae;D_5__Pantoea
3033e2e53ae9b1c88296c7198e9b2a0e	ADC	2.50	D_1__Proteobacteria;D_2__Alphaproteobacteria;D_3__Rhodobacterales;D_4__Rhodobacteraceae;D_5__Paracoccus;D_6__Paracoccus pantotrophus
071711647770cb3bf9d706aca604e7c6	ADC	3.43	D_1__Actinobacteria;D_2__Actinobacteria;D_3__Propionibacteriales;D_4__Nocardioidaceae;D_5__Nocardioides;D_6__uncultured bacterium
eb510b936a1f4a039fcac9c89ffbfba8	ADC	3.19	D_1__Firmicutes;D_2__Negativicutes;D_3__Selenomonadales;D_4__Veillonellaceae;D_5__Veillonella
268750ea351bdaef9526b245b577ba73	ADC	3.07	D_1__Proteobacteria;D_2__Gammaproteobacteria;D_3__Betaproteobacteriales;D_4__Burkholderiaceae;D_5__Ralstonia
3856f9150e7680da907bfea69604a7b1	ADC	3.41	D_1__Proteobacteria;D_2__Gammaproteobacteria;D_3__Oceanospirillales;D_4__Halomonadaceae;D_5__Halomonas
58eddb1e19ee5345168ea0707d2fd6c4	ADC	3.76	D_1__Proteobacteria;D_2__Gammaproteobacteria;D_3__Alteromonadales;D_4__Shewanellaceae;D_5__Shewanella
d581025ba0dfb782ea6ccec6c47c25cc	ADC	3.05	D_1__Bacteroidetes;D_2__Bacteroidia;D_3__Bacteroidales;D_4__Prevotellaceae;D_5__Prevotella 7;D_6__uncultured bacterium
e112d98f4f5a15ea1a8f6e0cf74442c7	ADC	2.97	D_1__Bacteroidetes;D_2__Bacteroidia;D_3__Bacteroidales;D_4__Porphyromonadaceae;D_5__Porphyromonas;D_6__uncultured bacterium
662d2fb98041b679c1ae0f5cca206ca0	ADC	4.35	D_1__Proteobacteria;D_2__Gammaproteobacteria;D_3__Oceanospirillales;D_4__Halomonadaceae;D_5__Halomonas
dbb28740f251c46e7ca4b8ffcce7e10a	ADC	3.70	D_1__Actinobacteria;D_2__Actinobacteria;D_3__Propionibacteriales;D_4__Propionibacteriaceae;D_5__Cutibacterium;D_6__uncultured bacterium
145153842c99cb7787c75295a5a4913b	ADC	2.85	D_1__Firmicutes;D_2__Bacilli;D_3__Lactobacillales;D_4__Lactobacillaceae;D_5__Lactobacillus;D_6__Lactobacillus sakei
8d56750cca83750b5c9d7af00f7c7397	ADC	3.59	D_1__Proteobacteria;D_2__Gammaproteobacteria;D_3__Oceanospirillales;D_4__Halomonadaceae;D_5__Halomonas
622a6674bc02878fb11ae72859cc3b63	ADC	2.99	D_1__Proteobacteria;D_2__Gammaproteobacteria;D_3__Alteromonadales;D_4__Shewanellaceae;D_5__Shewanella;D_6__Shewanella algae
f1fee2782a0741de587dc59fdf11900f	ADC	2.61	D_1__Firmicutes;D_2__Bacilli;D_3__Lactobacillales;D_4__Lactobacillaceae;D_5__Lactobacillus;D_6__Lactobacillus gasseri
e5a1064b590794d8e6df1c3a8eff2fd0	ADC	4.26	D_1__Proteobacteria;D_2__Gammaproteobacteria;D_3__Alteromonadales;D_4__Shewanellaceae;D_5__Shewanella;D_6__Shewanella algae
39a4f108f5d87a504f06c586d69b4124	ADC	2.83	D_1__Proteobacteria;D_2__Gammaproteobacteria;D_3__Oceanospirillales;D_4__Halomonadaceae;D_5__Halomonas

0483c565fea8c807a77875763baa962d	ADC	3.28	D_1__Actinobacteria;D_2__Actinobacteria;D_3__Micrococcales;D_4__Micrococcaceae;D_5__Arthrobacter
e2dd8469f34dac1f52f92cdc2f536468	ADC	3.02	D_1__Proteobacteria;D_2__Gammaproteobacteria;D_3__Oceanospirillales;D_4__Halomonadaceae;D_5__Halomonas
4436749ce5a8d869d57cc2d9f0b5a238	ADC	2.96	D_1__Bacteroidetes;D_2__Bacteroidia;D_3__Bacteroidales;D_4__Prevotellaceae;D_5__Prevotella;Ambiguous_taxa
084ed4d230fd1bb0c85cf552905de9d1	ADC	2.85	D_1__Proteobacteria;D_2__Gammaproteobacteria;D_3__Betaproteobacteriales;D_4__Burkholderiaceae;D_5__Ralstonia
e4ad5b79d54bfe23988e47c93911dc8f	ADC	2.82	D_1__Proteobacteria;D_2__Gammaproteobacteria;D_3__Oceanospirillales;D_4__Halomonadaceae;D_5__Halomonas

**ASV**	**Class**	**LDA score**	**Taxonomy**
1091b9ea4ea047f0df6c01ad9e4816ab	SRCC	3.79	D_1__Proteobacteria;D_2__Gammaproteobacteria;D_3__Pseudomonadales;D_4__Moraxellaceae;D_5__Acinetobacter;D_6__Acinetobacter calcoaceticus
290c7b043c03ada9bf916f901e0ae131	SRCC	4.03	D_1__Actinobacteria;D_2__Actinobacteria;D_3__Bifidobacteriales;D_4__Bifidobacteriaceae;D_5__Bifidobacterium;Ambiguous_taxa
f9298d25ff149c12090bef1cb2f4e3aa	SRCC	3.54	D_1__Proteobacteria;D_2__Gammaproteobacteria;D_3__Pseudomonadales;D_4__Pseudomonadaceae;D_5__Pseudomonas
6d8cb00243064ce7a3bd02f4499e84d7	SRCC	2.92	D_1__Proteobacteria;D_2__Gammaproteobacteria;D_3__Betaproteobacteriales;D_4__Burkholderiaceae;D_5__Ralstonia
f29fa9722e3d9ddb838b9e6e76b0fe95	SRCC	4.38	D_1__Proteobacteria;D_2__Gammaproteobacteria;D_3__Enterobacteriales;D_4__Enterobacteriaceae;D_5__Escherichia-Shigella
8211fef6ecdb7e3ae949b3341796c927	SRCC	3.09	D_1__Proteobacteria;D_2__Gammaproteobacteria;D_3__Xanthomonadales;D_4__Xanthomonadaceae;D_5__Stenotrophomonas
d8432d02d0697c77d832c03c2f1d9751	SRCC	3.30	D_1__Actinobacteria;D_2__Actinobacteria;D_3__Actinomycetales;D_4__Actinomycetaceae;D_5__Actinomyces
d12facd6bf98b141da05ad7984d31de8	SRCC	3.23	D_1__Actinobacteria;D_2__Actinobacteria;D_3__Propionibacteriales;D_4__Propionibacteriaceae;D_5__Cutibacterium;D_6__uncultured bacterium
62301505548ed6e72fd084bed78db070	SRCC	4.00	D_1__Bacteroidetes;D_2__Bacteroidia;D_3__Bacteroidales;D_4__Prevotellaceae;D_5__Prevotella 7;D_6__Prevotella sp. oral taxon 300
455ecdedb13d604f24ed12397cde2c6a	SRCC	2.65	D_1__Actinobacteria;D_2__Actinobacteria;D_3__Bifidobacteriales;D_4__Bifidobacteriaceae;D_5__Bifidobacterium;Ambiguous_taxa
4c0c7b14bb156f83de0c51ca691ed4ac	SRCC	2.89	D_1__Actinobacteria;D_2__Actinobacteria;D_3__Actinomycetales;D_4__Actinomycetaceae;D_5__Actinomyces;D_6__uncultured Actinomyces sp.
ca90d4bbe45126219cf290f3c27432a3	SRCC	4.40	D_1__Bacteroidetes;D_2__Bacteroidia;D_3__Bacteroidales;D_4__Prevotellaceae;D_5__Prevotella 7;D_6__Prevotella denticola
417b2e6855b9afd86c2ac9773ef0dee8	SRCC	3.76	D_1__BRC1
8da9879a8b0e49e8cc44b3b9726be28e	SRCC	2.95	D_1__Actinobacteria;D_2__Actinobacteria;D_3__Actinomycetales;D_4__Actinomycetaceae;D_5__Actinomyces
e6d8b1d04d9d90effe7a86d1e8838808	SRCC	4.09	D_1__Bacteroidetes;D_2__Bacteroidia;D_3__Bacteroidales;D_4__Prevotellaceae;D_5__Prevotella 7;D_6__Prevotella sp. oral taxon 300
44a396ed63474ccb4c419b8fea53df50	SRCC	3.82	D_1__Bacteroidetes;D_2__Bacteroidia;D_3__Bacteroidales;D_4__Prevotellaceae;D_5__Prevotella 7;D_6__Prevotella denticola

c90293b7e3e2f17cfc76ad0b9f5aba88	SRCC	2.77	D_1__Actinobacteria;D_2__Actinobacteria;D_3__Actinomycetales;D_4__Actinomycetaceae;D_5__Actinomyces;D_6__unidentified
d5d064158b57642d65f3be84d9ad9e93	SRCC	3.37	D_1__Firmicutes;D_2__Clostridia;D_3__Clostridiales;D_4__Lachnospiraceae;D_5__Roseburia;D_6__uncultured bacterium
da2b8313bb21bfc020f63fd96d26930f	SRCC	3.21	D_1__Proteobacteria;D_2__Gammaproteobacteria;D_3__Enterobacteriales;D_4__Enterobacteriaceae;D_5__Escherichia-Shigella
f7330e7d4661a841ed42cfa90aedbf33	SRCC	3.35	D_1__Proteobacteria;D_2__Gammaproteobacteria;D_3__Betaproteobacteriales;D_4__Burkholderiaceae;D_5__Ralstonia

Table S2. Details of Pathways reported in the circos-plot

Pathway	LDA score	Description	Classification
**ADC**			
PWY-5529	2.12	superpathway of bacteriochlorophyll a biosynthesis	Biosynthesis → Cofactor, Prosthetic Group, Electron Carrier, and Vitamin Biosynthesis → Porphyrin Compound Biosynthesis → Chlorophyll Biosynthesis → Chlorophyll a Biosynthesis
PWY-5913	2.16	TCA cycle VI (obligate autotrophs)	Generation of Precursor Metabolite and Energy
DHGLUCONATE-PYR-CAT-PWY	2.22	glucose degradation (oxidative)	Degradation/Utilization/Assimilation → Carbohydrate Degradation → Sugar Degradation
GLYCOLYSIS-E-D	2.29	superpathway of glycolysis and Entner-Doudoroff	Generation of Precursor Metabolite and Energy
PWY4FS-7	2.32	phosphatidylglycerol biosynthesis I (plastidic)	Biosynthesis → Fatty Acid and Lipid Biosynthesis → Phospholipid Biosynthesis → Phosphatidylglycerol Biosynthesis
PWY4FS-8	2.32	phosphatidylglycerol biosynthesis II (non-plastidic)	Biosynthesis → Fatty Acid and Lipid Biosynthesis → Phospholipid Biosynthesis → Phosphatidylglycerol Biosynthesis
TCA	2.33	TCA cycle I (prokaryotic)	Generation of Precursor Metabolite and Energy → TCA cycle
PWY-7197	2.34	pyrimidine deoxyribonucleotide phosphorylation	Biosynthesis → Nucleoside and Nucleotide Biosynthesis → Pyrimidine Nucleotide Biosynthesis → Pyrimidine Nucleotide Salvage
TRPSYN-PWY	2.37	L-tryptophan biosynthesis	Biosynthesis → Amino Acid Biosynthesis → Proteinogenic Amino Acid Biosynthesis → L-tryptophan Biosynthesis
PWY-5022	2.40	4-aminobutanoate degradation V	Degradation/Utilization/Assimilation → Amine and Polyamine Degradation → 4-Aminobutanoate Degradation
P105-PWY	2.40	TCA cycle IV (2-oxoglutarate decarboxylase)	Generation of Precursor Metabolite and Energy → TCA cycle
P101-PWY	2.40	ectoine biosynthesis	Biosynthesis → Amine and Polyamine Biosynthesis
GLYOXYLATE-BYPASS	2.42	glyoxylate cycle	Generation of Precursor Metabolite and Energy

ARGSYNBSUB-PWY	2.42	L-arginine biosynthesis II (acetyl cycle)	Biosynthesis → Amino Acid Biosynthesis → Proteinogenic Amino Acid Biosynthesis → L-arginine Biosynthesis
PWY-7400	2.43	L-arginine biosynthesis IV (archaebacteria)	Biosynthesis → Amino Acid Biosynthesis → Proteinogenic Amino Acid Biosynthesis → L-arginine Biosynthesis
ARGSYN-PWY	2.43	L-arginine biosynthesis I (via L-ornithine)	Biosynthesis → Amino Acid Biosynthesis → Proteinogenic Amino Acid Biosynthesis → L-arginine Biosynthesis
SULFATE-CYS-PWY	2.43	superpathway of sulfate assimilation and cysteine biosynthesis	Degradation/Utilization/Assimilation → Inorganic Nutrient Metabolism → Sulfur Compound Metabolism
PWY-5189	2.44	tetrapyrrole biosynthesis II (from glycine)	Biosynthesis → Cofactor, Prosthetic Group, Electron Carrier, and Vitamin Biosynthesis → Tetrapyrrole Biosynthesis
PWY0-1241	2.44	ADP-L-glycero-β-D-manno-heptose biosynthesis	Biosynthesis → Carbohydrate Biosynthesis → Sugar Biosynthesis → Sugar Nucleotide Biosynthesis → ADP-sugar Biosynthesis
FERMENTATION-PWY	2.45	mixed acid fermentation	Generation of Precursor Metabolite and Energy → Fermentation → Fermentation of Pyruvate → Pyruvate Fermentation to Ethanol
PENTOSE-P-PWY	2.45	pentose phosphate pathway	Generation of Precursor Metabolite and Energy → Pentose Phosphate Pathways
BRANCHED-CHAIN-AA-SYN-PWY	2.46	superpathway of branched amino acid biosynthesis	Biosynthesis → Amino Acid Biosynthesis
TCA-GLYOX-BYPASS	2.49	superpathway of glyoxylate bypass and TCA	Generation of Precursor Metabolite and Energy → TCA cycle
PWY0-1479	2.49	tRNA processing	Macromolecule Modification → Nucleic Acid Processing
PWY-5101	2.53	L-isoleucine biosynthesis II	Biosynthesis → Amino Acid Biosynthesis → Proteinogenic Amino Acid Biosynthesis → L-isoleucine Biosynthesis
UBISYN-PWY	2.53	superpathway of ubiquinol-8 biosynthesis (prokaryotic)	Biosynthesis → Cofactor, Prosthetic Group, Electron Carrier, and Vitamin Biosynthesis → Quinol and Quinone Biosynthesis → Ubiquinol Biosynthesis
AST-PWY	2.53	L-arginine degradation II (AST pathway)	Degradation/Utilization/Assimilation → Amino Acid Degradation → Proteinogenic Amino Acid Degradation → L-arginine Degradation
HISTSYN-PWY	2.55	L-histidine biosynthesis	Biosynthesis → Amino Acid Biosynthesis → Proteinogenic Amino Acid Biosynthesis → L-histidine Biosynthesis
SALVADEHYPOX-PWY	2.55	adenosine nucleotides degradation II	Degradation/Utilization/Assimilation → Nucleoside and Nucleotide Degradation → Purine Nucleotide Degradation → Adenosine Nucleotide Degradation
PYRIDOXSYN-PWY	2.56	pyridoxal 5'-phosphate biosynthesis I	Biosynthesis → Cofactor, Prosthetic Group, Electron Carrier, and Vitamin Biosynthesis → Vitamin Biosynthesis → Vitamin B6 Biosynthesis
PWY-5154	2.58	L-arginine biosynthesis III (via N-acetyl-L-citrulline)	Biosynthesis → Amino Acid Biosynthesis → Proteinogenic Amino Acid Biosynthesis → L-arginine Biosynthesis
GLUCOSE1PMETAB-PWY	2.61	glucose and glucose-1-phosphate degradation	Degradation/Utilization/Assimilation → Carbohydrate Degradation → Sugar Degradation

PWY-5857	2.62	ubiquinol-10 biosynthesis (prokaryotic)	Biosynthesis → Cofactor, Prosthetic Group, Electron Carrier, and Vitamin Biosynthesis → Quinol and Quinone Biosynthesis → Ubiquinol Biosynthesis
PWY-5856	2.62	ubiquinol-9 biosynthesis (prokaryotic)	Biosynthesis → Cofactor, Prosthetic Group, Electron Carrier, and Vitamin Biosynthesis → Quinol and Quinone Biosynthesis → Ubiquinol Biosynthesis
PWY-5855	2.62	ubiquinol-7 biosynthesis (prokaryotic)	Biosynthesis → Cofactor, Prosthetic Group, Electron Carrier, and Vitamin Biosynthesis → Quinol and Quinone Biosynthesis → Ubiquinol Biosynthesis
PWY-6708	2.62	ubiquinol-8 biosynthesis (prokaryotic)	Biosynthesis → Cofactor, Prosthetic Group, Electron Carrier, and Vitamin Biosynthesis → Quinol and Quinone Biosynthesis → Ubiquinol Biosynthesis
HEMESYN2-PWY	2.62	heme biosynthesis II (anaerobic)	Biosynthesis → Cofactor, Prosthetic Group, Electron Carrier, and Vitamin Biosynthesis → Porphyrin Compound Biosynthesis → Heme Biosynthesis → Heme b Biosynthesis
PWY-7328	2.65	superpathway of UDP-glucose-derived O-antigen building blocks biosynthesis	Biosynthesis → Carbohydrate Biosynthesis → Sugar Biosynthesis → Sugar Nucleotide Biosynthesis → UDP-sugar Biosynthesis
HEME-BIOSYNTHESIS-II	2.66	heme biosynthesis I (aerobic)	Biosynthesis → Cofactor, Prosthetic Group, Electron Carrier, and Vitamin Biosynthesis → Porphyrin Compound Biosynthesis → Heme Biosynthesis → Heme b Biosynthesis
GLUTORN-PWY	2.66	L-ornithine biosynthesis	Biosynthesis → Amino Acid Biosynthesis → Other Amino Acid Biosynthesis → L-Ornithine Biosynthesis
PWY-5384	2.67	sucrose degradation IV (sucrose phosphorylase)	Degradation/Utilization/Assimilation → Carbohydrate Degradation → Sugar Degradation → Sucrose Degradation
SO4ASSIM-PWY	2.69	sulfate reduction I (assimilatory)	Degradation/Utilization/Assimilation → Inorganic Nutrient Metabolism → Sulfur Compound Metabolism → Assimilatory Sulfate Reduction
PWY-7111	2.90	pyruvate fermentation to isobutanol (engineered)	Generation of Precursor Metabolite and Energy → Fermentation → Fermentation of Pyruvate
**SRCC**			
PWY-6397	-2.04	mycolyl-arabinogalactan-peptidoglycan complex biosynthesis	Biosynthesis → Cell Structure Biosynthesis → Cell Wall Biosynthesis
PWY-7371	-2.06	1,4-dihydroxy-6-naphthoate biosynthesis II	Biosynthesis → Cofactor, Prosthetic Group, Electron Carrier, and Vitamin Biosynthesis → Quinol and Quinone Biosynthesis → 1,4-dihydroxy-6-naphthoate biosynthesis
PWY-5304	-2.11	superpathway of sulfur oxidation (Acidianus ambivalens)	Degradation/Utilization/Assimilation → Inorganic Nutrient Metabolism → Sulfur Compound Metabolism → Sulfur Oxidation
PWY-6562	-2.16	norspermidine biosynthesis	Biosynthesis → Amine and Polyamine Biosynthesis
THREOCAT-PWY	-2.19	superpathway of L-threonine metabolism	Degradation/Utilization/Assimilation → Amino Acid Degradation → Proteinogenic Amino Acid Degradation → L-threonine Degradation

PWY-6612	-2.2	superpathway of tetrahydrofolate biosynthesis	Biosynthesis → Cofactor, Prosthetic Group, Electron Carrier, and Vitamin Biosynthesis → Vitamin Biosynthesis → Folate Biosynthesis
FOLSYN-PWY	-2.22	superpathway of tetrahydrofolate biosynthesis and salvage	Biosynthesis → Cofactor, Prosthetic Group, Electron Carrier, and Vitamin Biosynthesis → Vitamin Biosynthesis → Folate Biosynthesis
TEICHOICACID-PWY	-2.22	teichoic acid (poly-glycerol) biosynthesis	Biosynthesis → Cofactor, Prosthetic Group, Electron Carrier, and Vitamin Biosynthesis → Vitamin Biosynthesis → Folate Biosynthesis
PWY-6263	-2.23	superpathway of menaquinol-8 biosynthesis II	Biosynthesis → Cofactor, Prosthetic Group, Electron Carrier, and Vitamin Biosynthesis → Quinol and Quinone Biosynthesis → Menaquinol Biosynthesis Superpathways
PRPP-PWY	-2.23	superpathway of histidine, purine, and pyrimidine biosynthesis	Biosintesis pirimidine
P341-PWY	-2.24	glycolysis V (Pyrococcus)	Generation of Precursor Metabolite and Energy → Glycolysis
PWY-7392	-2.28	taxadiene biosynthesis (engineered)	Biosynthesis → Secondary Metabolite Biosynthesis → Terpenoid Biosynthesis → Diterpenoid Biosynthesis
PWY-7187	-2.29	pyrimidine deoxyribonucleotides de novo biosynthesis II	Biosynthesis → Nucleoside and Nucleotide Biosynthesis → 2'-Deoxyribonucleotide Biosynthesis → Pyrimidine Deoxyribonucleotide De Novo Biosynthesis
PWY-7377	-2.34	cob(II)yrinate a,c-diamide biosynthesis I (early cobalt insertion)	Biosynthesis → Cofactor, Prosthetic Group, Electron Carrier, and Vitamin Biosynthesis → cob(II)yrinate a,c-diamide biosynthesis
P162-PWY	-2.34	L-glutamate degradation V (via hydroxyglutarate)	Degradation/Utilization/Assimilation → Amino Acid Degradation → Proteinogenic Amino Acid Degradation → L-glutamate Degradation
LACTOSECAT-PWY	-2.36	lactose and galactose degradation I	Degradation/Utilization/Assimilation → Carbohydrate Degradation → Sugar Degradation → Galactose Degradation
PWY-6123	-2.37	inosine-5'-phosphate biosynthesis I	Biosynthesis → Nucleoside and Nucleotide Biosynthesis → Purine Nucleotide Biosynthesis → Purine Nucleotide De Novo Biosynthesis → Purine Riboucleotide De Novo Biosynthesis → Inosine-5'-phosphate Biosynthesis
PWY-922	-2.38	mevalonate pathway I	Biosynthesis → Secondary Metabolite Biosynthesis → Terpenoid Biosynthesis → Hemiterpene Biosynthesis → Isopentenyl Diphosphate Biosynthesis → Mevalonate Pathways
DENOVOPURINE2-PWY	-2.42	superpathway of purine nucleotides de novo biosynthesis II	Biosynthesis → Nucleoside and Nucleotide Biosynthesis → Purine Nucleotide Biosynthesis → Purine Nucleotide De Novo Biosynthesis
PWY-5989	-2.45	stearate biosynthesis II (bacteria and plants)	Biosynthesis → Fatty Acid and Lipid Biosynthesis → Fatty Acid Biosynthesis → Stearate Biosynthesis
PWY-6609	-2.45	adenine and adenosine salvage III	Biosynthesis → Nucleoside and Nucleotide Biosynthesis → Purine Nucleotide Biosynthesis → Purine Nucleotide Salvage → Adenine and Adenosine Salvage

PWY-5507	-2.46	adenosylcobalamin biosynthesis I (early cobalt insertion)	Biosynthesis → Cofactor, Prosthetic Group, Electron Carrier, and Vitamin Biosynthesis → Vitamin Biosynthesis → Cobamide Biosynthesis → Cobamide de novo Biosynthesis → Adenosylcobamide Biosynthesis → Adenosylcobalamin de novo Biosynthesis
PWY-5177	-2.46	glutaryl-CoA degradation	Degradation/Utilization/Assimilation → Carboxylate Degradation
PWY-5910	-2.47	superpathway of geranylgeranyldiphosphate biosynthesis I (via mevalonate)	Biosynthesis → Cofactor, Prosthetic Group, Electron Carrier, and Vitamin Biosynthesis → Polyprenyl Biosynthesis → Geranylgeranyl Diphosphate Biosynthesis; Biosynthesis → Secondary Metabolite Biosynthesis → Terpenoid Biosynthesis → Diterpenoid Biosynthesis
PWY-2941	-2.47	L-lysine biosynthesis II	Biosynthesis → Amino Acid Biosynthesis → Proteinogenic Amino Acid Biosynthesis → L-lysine Biosynthesis
PWY-6737	-2.49	starch degradation V	Degradation/Utilization/Assimilation → Carbohydrate Degradation → Polysaccharide Degradation → Starch Degradation
P381-PWY	-2.49	adenosylcobalamin biosynthesis II (late cobalt incorporation)	Biosynthesis → Cofactor, Prosthetic Group, Electron Carrier, and Vitamin Biosynthesis → Vitamin Biosynthesis → Cobamide Biosynthesis → Cobamide de novo Biosynthesis → Adenosylcobamide Biosynthesis → Adenosylcobalamin de novo Biosynthesis
CENTFERM-PWY	-2.53	pyruvate fermentation to butanoate	Generation of Precursor Metabolite and Energy → Fermentation → Fermentation of Pyruvate
GLCMANNANAUT-PWY	-2.59	superpathway of N-acetylglucosamine, N-acetylmannosamine and N-acetylneuraminate degradation	Degradation/Utilization/Assimilation → Amine and Polyamine Degradation
PWY-6590	-2.6	superpathway of Clostridium acetobutylicum acidogenic fermentation	Generation of Precursor Metabolite and Energy → Fermentation → Fermentation of Pyruvate
PWY-7456	-2.64	mannan degradation	Degradation/Utilization/Assimilation → Carbohydrate Degradation → Polysaccharide Degradation
PWY-6471	-2.65	peptidoglycan biosynthesis IV (Enterococcus faecium)	Biosynthesis → Cell Structure Biosynthesis → Cell Wall Biosynthesis → Peptidoglycan Biosynthesis
PWY-6588	-2.65	pyruvate fermentation to acetone	Generation of Precursor Metabolite and Energy → Fermentation → Fermentation of Pyruvate
P441-PWY	-2.67	superpathway of N-acetylneuraminate degradation	Degradation/Utilization/Assimilation → Carboxylate Degradation
PWY-5100	-2.67	pyruvate fermentation to acetate and lactate II	Degradation/Utilization/Assimilation → Carboxylate Degradation → Fermentation to Acetate → Pyruvate Fermentation to Acetate
REDCITCYC	-2.69	TCA cycle VIII (helicobacter)	Generation of Precursor Metabolite and Energy → TCA cycle
PWY-5971	-2.71	palmitate biosynthesis II (bacteria and plants)	Biosynthesis → Fatty Acid and Lipid Biosynthesis → Fatty Acid Biosynthesis → Palmitate Biosynthesis

P164-PWY	-2.81	purine nucleobases degradation I (anaerobic)	Degradation/Utilization/Assimilation → Nucleoside and Nucleotide Degradation → Purine Nucleotide Degradation
P42-PWY	-2.89	incomplete reductive TCA cycle	Degradation/Utilization/Assimilation → C1 Compound Utilization and Assimilation → CO2 Fixation → Autotrophic CO2 Fixation → Reductive TCA Cycles
P23-PWY	-3	reductive TCA cycle I	Degradation/Utilization/Assimilation → C1 Compound Utilization and Assimilation → CO2 Fixation → Autotrophic CO2 Fixation → Reductive TCA Cycles

Table S3. Relative abundance in microbial communities in SRCC, SRCC PNT, ADC and ADC PNT samples				
**Condition**	**SRCC**	**SRCC PNT**	**ADC**	**ADC PNT**
Proteobacteria	49.89%	48.16%	70.68%	65.72%
Firmicutes	18.36%	19.69%	12.22%	15.83%
Actinobacteria	13.29%	13.51%	11.36%	12.24%
Bacteroidetes	11.98%	13.50%	3.82%	4.05%
Fusobacteria	2.13%	1.40%	0.00%	0.30%
Patescibacteria	1.67%	0.60%	0.50%	0.30%
BRC1	0.97%	0.92%	0.00%	0.10%
Verrucomicrobia	0.49%	0.43%	0.08%	0.20%
Epsilonbacteraeota	0.42%	0.73%	0.06%	0.05%
Chloroflexi	0.27%	0.19%	0.05%	0.09%
Deinococcus-Thermus	0.19%	0.20%	0.06%	0.32%
other	0.18%	0.02%	0.03%	0.00%
Gemmatimonadetes	0.13%	0.06%	0.05%	0.14%
Cyanobacteria	0.03%	0.25%	0.04%	0.47%
Acidobacteria	0.00%	0.29%	0.72%	0.18%
Armatimonadetes	0.00%	0.06%	0.33%	0.00%
